# Psychiatric disorders in patients with rheumatoid arthritis

**DOI:** 10.1192/j.eurpsy.2024.466

**Published:** 2024-08-27

**Authors:** A. Feki, I. Sellami, I. Mnif, A. Abbes, Z. Gassara, S. Ben jemaa, M. Ezzeddine, M. H. Kallel, H. Fourati, R. Akrout, S. Baklouti

**Affiliations:** ^1^Rheumatology; ^2^occupational medicine, Hedi chaker hospital, Sfax, Tunisia

## Abstract

**Introduction:**

Rheumatoid arthritis (RA) is a systemic inflammatory disease that can lead to significant morbidity and especially to psychiatric disorders. Depression and anxiety are common symptoms in RA patients, and seem to influence disease activity, pain, and treatment response.

**Objectives:**

The aim of this study was to investigate the prevalence of depression and anxiety and their related factors in RA patients.

**Methods:**

100 patients diagnosed with rheumatoid arthritis according to ACR1987 or ACR/EULAR criteria 2010 were investigated. Demographic, clinical and laboratorial data were obtained from hospitals records.

The RA severity Disease Activity was assessed by the Disease Activity Score (DAS 28). Physical function was assessed by the Health Assessment Questionnaire-Disability Index (HAQ).

The Hospital Anxiety and Depression Scale (HAD a/d) was used to evaluate the depression and anxiety symptoms. Patients with results greater than 11 are considered depressed or anxious.

**Results:**

The group studied included 87% of women and 13% of men. The median age was 55.2 years [27-83]. The median disease duration was 11.7± 8.9 years.

The majority of the patients were unemployed, they were housewives in 65% of the cases, retired in 6% and 2 % had taken sick leave.

The median ESR was 44 ± 31.6 mm, and the median of CRP level was 26 ± 35.3 mg/l. The median disease activity (DAS 28) was 4.6 ± 1.4. Forty-five % had moderate disease activity (3.2 ≤DAS 28 ≤ 5.1), and 27% of the sample had high disease activity (DAS 28 > 5.1). The mean of the HAQ was 1.1 [0-3].

The anxiety and depression questionnaire (HAD a/d) showed means of 10.6 for depression, and 10.25 for anxiety respectively. Depression was presented in 46 % of RA patients. Anxiety was presented in 48 %.

There was a correlation of HAD a/d with employment status (p<0.05), and functional disability (HAQ) (p<0.001).

There was no significant association of anxiety and depression scales with RA disease activity.

**Conclusions:**

Chronic inflammation impairs the physiological responses to stress, resulting in depression, anxiety which leads to a worse long-term outcome in RA.

Physical disability and social factors, are predictive of psychiatric disorders in RA. This fact must be taken into account when evaluating therapeutic response.

**Disclosure of Interest:**

None Declared

